# The hormone regulatory mechanism underlying parthenocarpic fruit formation in tomato

**DOI:** 10.3389/fpls.2024.1404980

**Published:** 2024-07-25

**Authors:** Hongling Guan, Xiaolong Yang, Yuxiang Lin, Baoxing Xie, Xinyue Zhang, Chongjian Ma, Rui Xia, Riyuan Chen, Yanwei Hao

**Affiliations:** ^1^ College of Horticulture, South China Agricultural University, Guangzhou, China; ^2^ Guangdong Provincial Key Laboratory of Utilization and Conservation of Food and Medicinal Resources in Northern Region, School of Biology and Agriculture, Shaoguan University, Shaoguan, China; ^3^ Guangdong Key Laboratory for New Technology Research of Vegetables, Vegetable Research Institute, Guangdong Academy of Agricultural Sciences, Guangzhou, China

**Keywords:** tomato, fruit set, parthenocarpy, phytohormones, seedless

## Abstract

Parthenocarpic fruits, known for their superior taste and reliable yields in adverse conditions, develop without the need for fertilization or pollination. Exploring the physiological and molecular mechanisms behind parthenocarpic fruit development holds both theoretical and practical significance, making it a crucial area of study. This review examines how plant hormones and MADS-box transcription factors control parthenocarpic fruit formation. It delves into various aspects of plant hormones-including auxin, gibberellic acid, cytokinins, ethylene, and abscisic acid—ranging from external application to biosynthesis, metabolism, signaling pathways, and their interplay in influencing parthenocarpic fruit development. The review also explores the involvement of MADS family gene functions in these processes. Lastly, we highlight existing knowledge gaps and propose directions for future research on parthenocarpy.

## Introduction

Tomato is one of the world’s most significant vegetable crops due to its economic and nutritional importance. Tomato fruit development is traditionally divided into five stages (Ezura et al., 2023). Stage I involves flower maturation before pollination and fertilization occur. Stage II spans from fertilization to four days post-anthesis, marking the fruit set phase. Stages III through V encompass fruit growth and ripening. Successful fruit development hinges on pollination and fertilization, which are susceptible to extreme environmental conditions such as high or low temperatures (Picken, 1984; Mesihovic et al., 2016). However, parthenocarpy, the development of fruit without fertilization resulting in seedless fruits, can adapt well to unfavorable conditions (Gorguet et al., 2005).

Reports of parthenocarpy date back to the 1890s. This phenomenon is prevalent among many horticultural crops such as tomato (Molesini et al., 2020; Sharif et al., 2022), cucumber (Gou et al., 2022), eggplant (Zhou et al., 2023), pumpkin (Luo et al., 2021) and holds significant agricultural value. Parthenocarpy comes in two main forms: stimulative and natural (Chen et al., 2001; Zhu et al., 2007). Stimulative parthenocarpy can be induced through methods like hand-stripping, pollination with sterile pollen or chemical treatments; however, it’s not inheritable. In contrast, natural parthenocarpy is genetically determined and subdivides into obligate and facultative types. Obligate parthenocarpy consistently yields seedless fruits regardless of conditions, while facultative parthenocarpy does so only when conditions are unfavorable—otherwise it can produce seeded fruits with normal pollination (Mazzucato et al., 1999; Varoquaux et al., 2000).

In plants, auxins and gibberellins are key hormones regulating parthenocarpy (Pascual et al., 2009; Mignolli et al., 2015), with auxins functioning upstream of gibberellins (Serrani et al., 2008). Parthenocarpic tomato varieties exhibit significantly higher levels of IAA (indole-3-acetic acid) and GA_3_ (gibberellic acid) in the ovaries during pre-flowering and flowering stages compared to non-parthenocarpic types (Hazra et al., 2010). Treatment with either hormone (IAA or GA_3_) can promote ovary development, resulting in seedless fruits (Serrani et al., 2008; Niu et al., 2024). Other hormones like cytokinins, abscisic acid (ABA), and ethylene also contribute to parthenocarpic fruit development (Ding et al., 2013; Shinozaki et al., 2015; Kai et al., 2019). Cytokinins are vital for cell division and early fruit growth, especially after pollination and fertilization (Ding et al., 2013). Applying CPPU (N-(2-chloro-4-pyridyl)-N’-phenylurea) during flowering can induce seedless tomato fruits (Ding et al., 2013). For parthenocarpic plants, ABA inhibit fruit set and fruit growth but not the growth of plants (Rodrigo and García-Martínez, 1998). Significant decrease in abscisic acid content was found in tomatoes ovaries after completion of pollination and fertilization or treatment with auxin (Mariotti et al., 2011). Ethylene also plays a role: ACC (1-aminocyclopropane-1-carboxylic acid) application hinders fruit set while using an ethylene receptor inhibitor like 1-MCP (1-methylcyclopropene) on unpollinated ovaries encourages parthenocarpy (An et al., 2020). However, external hormone treatments do not affect heredity and may cause malformed fruits or prevent the proper opening of subsequent flowers, ultimately leading to low-quality hollow fruits (Abad and Monteiro, 1989). Advances in genetic engineering have identified genes involved in hormonal synthesis, transport, and metabolism that can induce stable inheritable parthenocarpy in tomatoes (Olimpieri et al., 2007; de Jong et al., 2011; Mignolli et al., 2015).

Parthenocarpy is a valuable trait in horticulture, playing a crucial role in production practices, particularly in controlled cultivation environments where pollination is limited. Extreme weather conditions such as high temperatures with humidity or cold temperatures with low light can severely impact tomato pollen development, resulting in reduced fruit yield and quality (Pan et al., 2021). For growers, the parthenocarpic characteristic eliminates the need for manual labor, bee pollination, and external growth regulators, ensuring consistent yields and lowering production costs (Chen et al., 2017; Knapp et al., 2017). Consumers and processors often prefer seedless fruits; thus, parthenocarpy enhances the marketability of horticultural products (Pandolfini et al., 2002; Knapp et al., 2017). Unraveling the molecular mechanisms behind parthenocarpic fruit development will provide deeper insights at the molecular level, facilitating the study of this phenomenon and establishing a robust theoretical basis for breeding parthenocarpic varieties.

## Understanding the role of plant hormones in parthenocarpy

Typically, fruits form through pollination and fertilization. However, seedless fruits can develop from unfertilized ovaries when plant growth regulators are applied during flowering. Studies show that gibberellins, auxins, and cytokinins have the capacity to promote parthenocarpy, whereas abscisic acid and ethylene have inhibitory effects on this process (Maroto et al., 2005; Nitsch et al., 2009; Shinozaki et al., 2015; Kai et al., 2019; Molesini et al., 2020; Niu et al., 2024).

## Understanding the molecular mechanisms behind auxin-induced parthenocarpy

Auxin is essential for regulating plant growth, development, and fruit setting. Studies show that auxin levels rise significantly in ovaries after successful pollination and fertilization (Gillaspy et al., 1993; Zhang et al., 2021). Parthenocarpic tomato varieties naturally have higher auxin concentrations in their ovaries compared to normal fruits, enabling them to develop without fertilization (Qiu, 1984; Gorguet et al., 2005; Zhang et al., 2021). Applying exogenous auxin to unfertilized ovaries can also induce parthenocarpy (Nitsch et al., 2009). Furthermore, enhancing the expression of genes related to auxin production can trigger parthenocarpy and promote fruit development in tomatoes (Rotino et al., 1997; Matsuo et al., 2020). Within 48 hours after pollination, there’s an upsurge of auxin-responsive genes, resulting in the activation of auxin signaling (Vriezen et al., 2008). Transcriptomic analyses reveal that expanding locular cells in pollinated fruits predominantly express genes associated with synthesis, transport, and response to auxin (Lemaire-Chamley et al., 2005).

Indole-3-acetic acid (IAA) is the most common phytohormone of the auxin class. It is synthesized through both tryptophan-dependent and independent pathways (Jahn et al., 2021). Recent discoveries have unveiled numerous catalytic enzymes and key regulatory genes involved in the tryptophan-dependent pathway for auxin production, while the alternative pathway remains less understood. Consequently, research on the tryptophan-dependent route is more advanced (Zhao, 2010). Researchers typically categorize the tryptophan-dependent pathways into four branches based on their main intermediates: indole-3-pyruvic acid (IPyA), tryptamine (TAM), indole-3- acetaldoxime/indole-3-acetonitrile (IAOx-IAN), and indole-3-acetamide (IAM) (Zhao, 2010; Jahn et al., 2021). Studies reveal that Agrobacterium tumefaciens’ *iaaM* gene converts tryptophan to IAM, which then hydrolyzes to IAA, promoting local IAA synthesis (Gaudin and Jouanin, 1995). To investigate local auxin production’s impact on fruit development, researchers used the placenta- and ovule-specific promoter *DefH9* to drive targeted expression of *iaaM* in tomatoes (**Table 1**). This initial discovery provided insights into the direct impact of auxin on fruit growth, leading to the development of transgenic plants with parthenocarpic abilities (Rotino et al., 1997). Similar outcomes with parthenocarpic fruits were observed in other genetically modified species like raspberries and strawberries expressing specific *iaaM* (**Table 1**) (Mezzetti et al., 2004; Yin et al., 2006; Costantini et al., 2007). In *Solanaceae* plants, studies found that a naturally occurring parthenocarpic mutant *pad-1* exhibited elevated auxin levels within its ovaries. The non-functional allele *pad-1* was identified as pivotal for this trait (**Table 1**) (Matsuo et al., 2020); it normally facilitates IPyA conversion to Trp in eggplant ovaries, thus restraining *de novo* IAA synthesis. *Pad-1’s* function appears critical for preventing excessive IAA buildup in unfertilized ovaries (Matsuo et al., 2020).

**Table 1:** Genes associated with hormone regulation in tomato parthenocarpy.

Underlying pathway	Gene	ID	Cause	References
Auxin biosynthesis genes	DefH9-iaaM		overexpressing	Ficcadenti et al., 1999
	PAD-1	Solyc03g120450	silencing	Matsuo et al., 2020
Auxin transport genes	PIN4	Solyc05g008060	silencing	Mounet et al., 2012
	PIN8	Solyc02g087660	silencing	Gan et al., 2019
	AUCSIA-1	Solyc10g054660	silencing	Molesini et al., 2009
	AUCSIA-2	Solyc01g110540	silencing	Molesini et al., 2009
Auxin receptor and signaling transduction genes	TIR1	Solyc09g074520	overexpressing	Ren et al., 2011
	IAA9	Solyc04g076850	silencing	Wang et al., 2005; Zhang et al., 2007
	ARF5	Solyc04g081240	silencing	Liu et al., 2018c
	ARF7	Solyc07g042260	silencing	de Jong et al., 2009
	ARF8	Solyc03g031970	silencing	Goetz et al., 2006
Gibberellin biosynthesis genes	GA20ox1	Solyc03g006880	overexpressing	García-Hurtado et al., 2012
Gibberellin metabolism genes	GA2ox1	Solyc05g053340	silencing	Martínez-Bello et al., 2015
	GA2ox2	Solyc07g056670	silencing	Martínez-Bello et al., 2015
	GA2ox3	Solyc01g079200	silencing	Martínez-Bello et al., 2015
	GA2ox4	Solyc07g061720	silencing	Martínez-Bello et al., 2015
	GA2ox5	Solyc07g061730	silencing	Martínez-Bello et al., 2015
Gibberellin signaling transduction genes	DELLA	Solyc11g011260	silencing	Martí et al., 2007
Ethylene receptor and signaling transduction genes	ETR1	Solyc12g011330	silencing	Shinozaki et al., 2015
	TPR1	Solyc07g006180	overexpressing	Lin et al., 2008
	EIN2	Solyc09g007870	silencing	Zhu et al., 2006
Abscisic acid biosynthesis genes	NCED1	Solyc07g056570	overexpressing	Kai et al., 2019
Cytokinin biosynthesis genes	IPT		overexpressing	Mao et al., 2002

Auxin transport, which is essential for plant growth and development, involves both long-distance and short-range movement through cell membranes (Teale et al., 2006; Hammes et al., 2022). Key players in this process are the auxin transporters: the PIN-FORMED (PIN), AUXIN1/LIKE-AUX1 (AUX/LAX), and ATP-binding cassette subfamily B/multidrug resistance/phosphoglycoprotein (ABCB/MDR/PGP) families (Zazimalova et al., 2010). These membrane proteins reside on the plasma or intracellular membranes. The AUX/LAX transporters facilitate incoming auxin flow, whereas the PIN and ABCB families mainly handle outgoing flux (Zazimalova et al., 2010). Most PIN proteins are strategically positioned on cell membranes to direct precisely the polar transport of auxin (Krecek et al., 2009; Adamowski and Friml, 2015). Blocking this directional transport with NPA (N -1-naphthylphthalamic acid)—an inhibitor—during tomato flowering can induce parthenocarpy (Serrani et al., 2010). *SlPIN4* of the PIN family plays an important role in auxins regulation of fruit set in tomato; silencing it causes the development of seedless fruits (**Table 1**) (Mounet et al., 2012). Additionally, *SlPIN8* silencing not only affects the vegetative growth of tomato, but also severely affects pollen development and ultimately leads to parthenocarpic fruits (**Table 1**) (Gan et al., 2019). Lastly, research reveals that targeting *Aucsia*—a gene family implicated in tomato fruit regulation—via RNAi technology spurs seedless fruit formation while dramatically amplifying IAA levels within flower buds before bloom initiation (**Table 1**) (Molesini et al., 2009).

**Figure 1:**
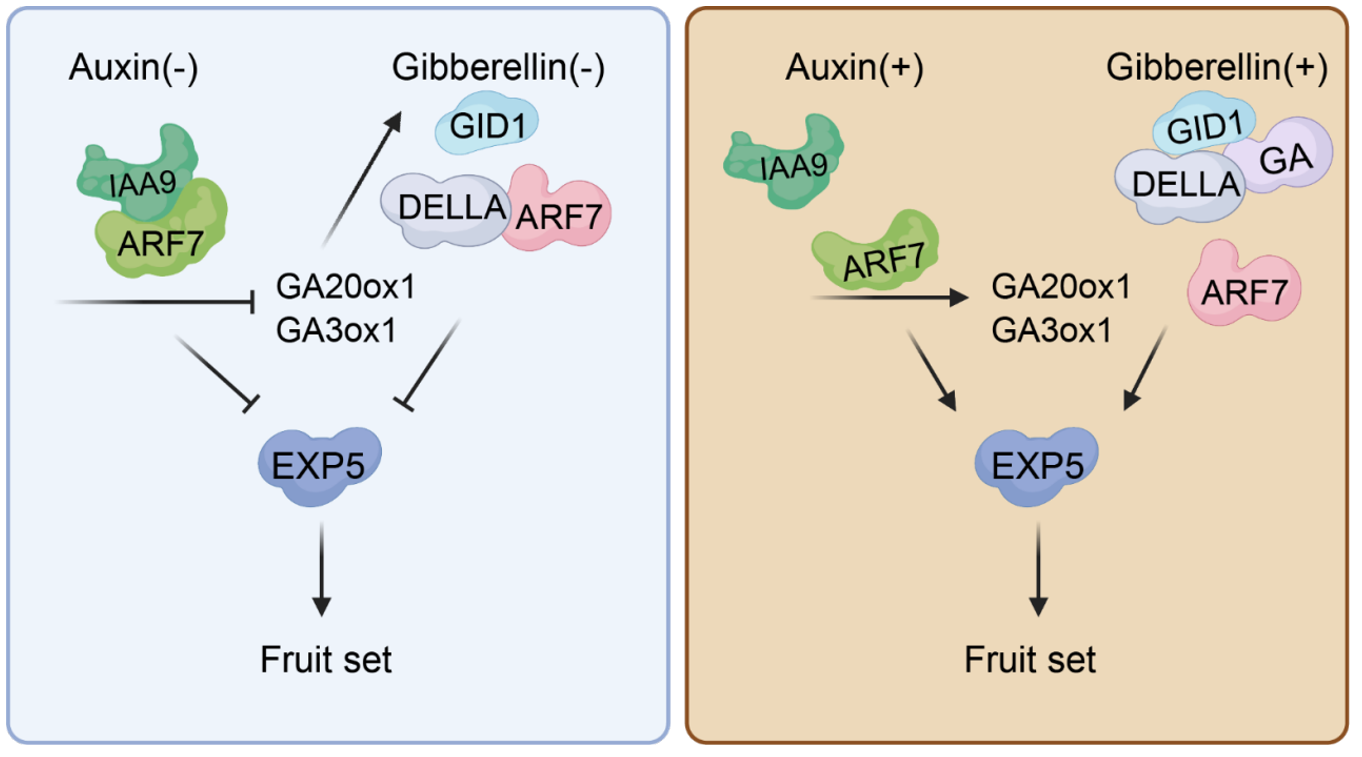
Auxin and gibberellin interactions in tomato fruit set. Prior to pollination, the SlARF7/SlIAA9 complex acts as an inhibitor of auxin signaling, while the DELLA protein binds with ARF7 when both auxin and gibberellin levels are low. Together, these proteins suppress tomato fruit set by downregulating EXP5 expression. The SlARF7/SlIAA9 complex also represses genes responsible for gibberellin production (GA20ox1 and GA3ox1), resulting in reduced gibberellin content. Following pollination, increased auxin within the fertilized ovule leads to IAA9 breakdown. Concurrently, higher gibberellin levels facilitate DELLA degradation. With both DELLA and SlIAA9 removed, ARF7 activates specific genes responsive to auxins that encourage fruit set.

The auxin receptor is essential for recognizing auxin, allowing it to bind and initiate several downstream reactions (Mockaitis and Estelle, 2008). Auxin facilitates the direct interaction between the Aux/IAA transcriptional repressor proteins and the TIR1/AFB auxin receptors. This binding encourages the breakdown of Aux/IAA proteins, which lifts suppression on ARF transcription factors and activates plant auxin signaling (Leyser, 2018). The TIR1/AFB protein, a critical element in this pathway, occupies a pivotal role. As a member of the F-box gene family, *TIR1* encodes for the auxin receptor protein. Overexpression of *SlTIR1* affects floral organ formation and results in parthenocarpy in tomatoes (**Table 1**) (Ren et al., 2011). *PslTIR1*, the homologous *TIR1* in plum, was also found to induced parthenocarpic fruits formation in tomato (El-Sharkawy et al., 2016). Additionally, studies show that auxin signal transduction is a complex process governed by multiple factors and pathways. Shortly after exposure to auxin, there’s a notable increase in the expression of early auxin response genes, categorized into three families: *SAUR*, *GH3*, and *Aux/IAA* (Luo et al., 2018). The Aux/IAA family plays a pivotal role in gene regulation following auxin exposure. It interacts with auxin response factors (ARFs), forming dimers that inhibit ARFs’ transcriptional regulatory functions (Leyser, 2018). ARFs are specialized transcription factors binding to the AuxRE (TGTCTC) sequence in early auxin-response gene promoters to regulate their expression (Ulmasov et al., 1995). Research indicates that both *ARFs* and *Aux/IAAs* contribute to tomato fruit development and parthenocarpy. Reducing *SlIAA9* expression results in a pleiotropic phenotype. It has simple leaves and fruit development is triggered before fertilization. This rapid enlargement of the ovary leads to the distancing of the stigma from stamens, thereby disrupting self-pollination and favoring the development of seedless fruit (Wang et al., 2005). Similarly, tomatoes with the *SlIAA9* loss-of-function mutant ‘*entire*’ show parthenocarpic traits but retain wild-type appearance (**Table 1**) (Hu et al., 2023). Furthermore, *IAA9* has been extensively studied and modified by cutting-edge technologies (Ueta et al., 2017; Mubarok et al., 2023). The combined action of *ARF7* and *IAA9* regulates parthenocarpy in tomatoes; double mutants display an even more pronounced phenotype (**Figure 1**) (Hu et al., 2018). Compared to the low expression levels in tomato pollinated ovaries, *SlARF5* displays high levels in ovaries under emasculation. Silencing *SlARF5* results in seedless fruits post-emasculation (**Table 1**) (Liu et al., 2018c). Transgenic tomatoes expressing reduced levels of *SlARF7* develop heart-shaped fruits with thicker skins due to cell expansion—a sign of parthenocarpy as well (de Jong et al., 2009). *SlARF8* disrupts post-fertilization induction of fruit and seed development by inhibiting carpel development (Goetz et al., 2006). *slarf8A*, *slarf8B* mutant combinations produced seedless parthenocarpic fruits (Hu et al., 2023; Israeli et al., 2023). *slymiR167*-*SlARF8A/B-SlGH3.4* is an important regulatory module during the development of locular and placenta tissues of tomato fruits (Hua et al., 2024). ERECTA (ER) is a receptor-like kinase (RLK) family protein known for its involvement in diverse developmental processes. It modulates fruit development via auxin signaling in tomato (Chen et al., 2024).

## Understanding the molecular mechanisms behind gibberellin-induced parthenocarpy

Gibberellin is a tetracyclic triterpenoid compound, with over a hundred types identified in various organisms (Hedden, 2020). Gibberellins stimulate cell division and growth, initiate seed germination, contribute to determining plant sex ratios, and can induce the formation of seedless fruit (Gao and Chu, 2020; Mäkilä et al., 2023). The parthenocarpic fruit (*pat*) gene is a recessive mutation enabling parthenocarpy, producing seedless fruits without pollination and fertilization (Beraldi et al., 2004). Studies on parthenocarpic tomato varieties with *pat*, *pat-2* and *pat-3/pat-4* genotypes reveal that gibberellin biosynthesis plays a crucial role during early fruit development (**Table 2**). In *pat-2* ovaries, the levels of GA_20_ rise significantly, while those of GA_19_ fall. Conversely, in varieties carrying the *pat-3/pat-4* genotype, activation of the gibberellin hydroxylation pathway occurs earlier (Fos et al., 2000).

**Table 2:** Germplasm resources of parthenocarpy in tomatoes.

Gene	Gene on chromosome	Variety	References
pat (HB15A)	chr3	Montfavet 191	Beraldi et al., 2004
pat-2	chr4	Severianin	Lin et al., 1984
pat-3/pat-4	chr4	RP75/79	Pascual et al., 2009
pat4.1	chr4	IL5-1	Gorguet et al., 2008
pat4.2	chr4	IVT-line 1	Gorguet et al., 2008
pat5.1	chr5	IL5-1	Gorguet et al., 2008
pat9.1	chr9	IVT-line 1	Gorguet et al., 2008
pat-k (AGL6)	chr1	MPK-1	Takisawa et al., 2018

Plant gibberellin production involves mevalonate (MVA) transitioning through plastids and endoplasmic reticulum to yield kaurene intermediates via enzymes; these then convert into various forms of gibberellins (GAs) in the cytoplasm thanks to enzymes like GA20 oxidase (GA20oxs), GA3 oxidase (GA3oxs), and GA2 oxidase (GA2oxs) (Hedden, 2020). Research indicates that enzymatic activity from *GA2oxs* inhibits seedless fruit development or parthenocarpy, both *GA20oxs* and *GA3oxs* endorse it (Wang et al., 2020). In tomatoes, overexpression of genes related to *GA20oxs* increases the plant’s natural gibberellin levels, leading to seedless fruit formation. Tomatoes with parthenocarpic ability display elevated expression of genes associated with *GA20oxs* and *GA3oxs*, whereas those linked to *GA2oxs* are less expressed (García-Hurtado et al., 2012; Martínez-Bello et al., 2015; Okabe et al., 2019; Takei et al., 2019). Additionally, external hormone application can activate the GA signaling pathway, promote gene expression related to both *GA20oxs* and *GA3oxs*, while reducing gene expression related to *GA2oxs*, thereby increasing the level of gibberellins within plants to induce parthenocarpy (Gorguet et al., 2005; Liu et al., 2018b; Cong et al., 2019).

Without fertilization, applying gibberellins externally can induce parthenocarpy by activating the GA signaling pathway (Gorguet et al., 2005). The gibberellic acid insensitive (GAI), repressor of GAI (RGA), and scarecrow (SCR) (GRAS) family is a class of transcription factors crucial for plant responses to adversity, stress, and aspects of growth and development. DELLA proteins are involved in parthenocarpy and serve as negative regulators of the GA response (McGinnis et al., 2003; Ueguchi-Tanaka et al., 2005; Murase et al., 2008). The breakdown of these proteins is a key step in propagating GA signals (Ito et al., 2018). *Arabidopsis thaliana* has five *DELLA* genes. Mutants lacking these five genes show similar seedless fruit traits underlining the importance of the GA signaling pathway in fruit development without fertilization (Dorcey et al., 2009; Fuentes et al., 2012). In tomato plants, mutants with an altered *SlDELLA* gene (*PROCERA*) and those with reduced expression via RNA interference can develop seedless fruits (Martí et al., 2007; Carrera et al., 2012; Livne et al., 2015). However, *SlDELLA* RNAi tomatoes produce smaller and noticeably elongated fruits compared to normal ones due to reduced circumference while maintaining width (**Table 1**) (Martí et al., 2007). Furthermore, GA signaling factor *SlMYB33*, which was depressed by GA treatment, induced parthenocarpic fruit set in tomato (Niu et al., 2024).

## Understanding the molecular mechanisms behind other hormone-induced parthenocarpy

Cytokinin, a plant hormone, stimulates cell division and differentiation in plant growth and contributes to parthenocarpy (Vivian-Smith and Koltunow, 1999; Mok and Mok, 2001). Following successful pollination and fertilization, cytokinins accumulate significantly, playing a crucial role in the initial phases of fruit development. Applying CPPU and 2,4-D to flowering watermelons can boost fruit set rates without negatively impacting fruit quality (Maroto et al., 2005). Initially used to induce seedless grapes, CPPU has been shown to produce seedless grapes in 24% to 44% of cases (Iwahori et al., 1988). While cytokinins can induce parthenocarpy in various crops, they are primarily used in cucurbit crops (Su et al., 2021). As early as 1955, Skoog and Miller first discovered agonist (kinetin, KT), followed by zeatin (ZT), isopentenyl adenine (iP), and others cytokinins (Miller et al., 1955). The key enzymes for cytokinin synthesis, isopentenyl-transferases (IPTs), were first found in Agrobacterium rhizogenes (Zhang, 2013; Nguyen et al., 2021). In cucumbers, *CsIPT2* expression is higher in parthenocarpic fruit than in pollinated fruit, indicating its significant role in cucumber parthenocarpy regulation (Zhang, 2013). Moreover, *IPT* overexpression in tomatoes has been shown to increase cytokinin levels and produce parthenocarpic fruit (**Table 1**) (Mao et al., 2002).

Ethylene, a pivotal plant hormone, primarily governs parthenocarpy by suppressing fruit set and interacting with other hormones. Reduced ethylene levels in the ovary facilitate parthenocarpy. Yet, when pollinated tomatoes receive treatment with ACC, an ethylene precursor, it may trigger fruit dropping (Shinozaki et al., 2015). The *iaa9–3* tomato mutant, which is naturally parthenocarpic, exhibits a decreased in ethylene levels similar to the reduction observed in normal tomatoes post-pollination. Introducing the ethylene inhibitor 1-MCP to unpollinated tomatoes can induce parthenocarpy (Shinozaki et al., 2015); fruits resulting from this resemble those of the *sletr1–1* mutant which is insensitive to ethylene and show elongation along with notable cell enlargement (**Table 1**) (Shinozaki et al., 2015).

The pathway for synthesizing ethylene chiefly involves S-adenosylmethionine (SAM) synthase, 1-aminocyclopropane-1-carboxylic acid (ACC) synthase, and ACC oxidase (ACO) (Yang and Hoffman, 1984). Ethylene’s biological role hinges on its signal transduction path: initiated by ETR family receptor detection and conveyed through CTR kinases as well as EIN3/EILs until activating downstream responders like the ERF family genes—which then modulate further gene expression. While regulators within both the ETR family and CTR kinase group predominantly mediate negative feedback for ethylene responses, elements such as *EIN2* alongside downstream actors like EIN3/EILs champion positive control (**Table 1**) (Mata et al., 2018; Ma and Dong, 2021). SlTPR1 can interact with ethylene receptors NR and LeETR1. Overexpression of *SlTPR1* can result in parthenocarpic fruits and also lead to the formation of abnormal and sterile flowers. Overexpression of *SlTPR1* in Arabidopsis can generate similar phenotypes (Lin et al., 2008). Silencing of *LeEIN2* in plants leads to delayed fruit development and ripening, as well as a reduced number of seeds compared to the wild type, resulting in a phenotype similar to parthenocarpy (Zhu et al., 2006).

Abscisic acid (ABA) impedes plant growth, particularly in parthenocarpic plants where it curtails both growth and their ability to develop fruit without fertilization (Rodrigo and García-Martínez, 1998). In tomatoes, ABA levels drop notably after pollination or auxin treatment (Mariotti et al., 2011). The synthesis of ABA is primarily regulated by the enzyme 9-cis-epoxycarotenoid dioxygenase (NCED), which converts 9-cis-epoxycarotenoid into the C15 precursor of ABA, zeaxanthin (Schwartz et al., 1997). A marked increase in ABA concentration and *NCED1* expression occurs in tomato ovaries during the three days leading up to and including flowering. Overexpressing *SlNCED1* also raises ABA levels, disrupting hormonal balance in the ovary and leading to parthenocarpic fruit production (**Table 1**) (Kai et al., 2019).

Recent studies have revealed that other hormones like polyamines (PA), melatonin (MT), and brassinolide (BR) can provoke parthenocarpy as well (Fos et al., 2003; Liu et al., 2018a). Chalcone synthase (*CHS*) gene initiates flavonoid biosynthesis. However, when this gene is silenced by RNAi in tomatoes, we observed not only a decrease in total flavonoid content, transcription levels of *chs1* and *chs2* genes, and CHS activity, but also the occurrence of parthenocarpic fruits (**Table 3**) (Schijlen et al., 2007).

**Table 3:** Additional genes regulating tomato parthenocarpy.

Family	Gene	ID	Cause	References
R2R3 MYB	GAMYB1	Solyc01g009070	silencing	da Silva et al., 2017
R2R3 MYB	GAMYB2	Solyc06g073640	silencing	da Silva et al., 2017
TOPLESS	TPL1	Solyc03g117360	silencing	He et al., 2021
HD-ZipIII	HB15A	Solyc03g120910	silencing	Clepet et al., 2021
DOF	DOF10	Solyc02g090310	silencing	Rojas-Gracia et al., 2019
CYP78A	KLUH	Solyc03g114940	overexpressing	Gupta et al., 2021
rol	rolB		overexpressing	Shabtai et al., 2007
high fruit set under stress	HFS		silencing	Meco et al., 2019
Arlequin	Alq		silencing	Ribelles et al., 2019
SPL/NZZ	HYDRA	Solyc07g063670	silencing	Rojas-Gracia et al., 2017
F-box	HWS	Solyc01g095370	silencing	Damayanti et al., 2019
chalcone synthase	CHS	Solyc09g091510	silencing	Schijlen et al., 2007
chalcone synthase	CHS	Solyc05g053550	silencing	Schijlen et al., 2007
ent-copalyl diphosphate synthase	CPS	Solyc06g084240	silencing	Hu et al., 2018
small parthenocarpic fruit and flower	SPFF	Solyc04g077010	silencing	Takei et al., 2019

## The interplay of various hormones in parthenocarpy

Parthenocarpy can only be induced when plant hormones are balanced. If the level of any hormone in the ovary is too high or too low, it hinders parthenocarpy (Chen et al., 2001). Research shows that a mix of hormones more effectively induces parthenocarpy than a single hormone, which often results in misshapen fruit and reduced quality. For instance, using just CPPU or gibberellin (GA_3_) to induce parthenocarpy in tomatoes leads to smaller fruits (Matsuo et al., 2012). Using only auxin (indole-3-acetic acid, IAA) produces thicker-skinned fruits compared to those from pollination. However, applying 2000 mg of GA_3_ with 2–20 ng of synthetic auxin 2,4-D yields fruits comparable in size and shape to naturally pollinated ones (Serrani et al., 2007). A combination of gibberellin and cytokinin also creates normal-sized fruits with increased weight compared to those induced by CPPU or GA_3_ alone (Ding et al., 2013). In fruit trees like pears, using GA_4 + 7_ alone elongates the fruit; however, adding multiple hormones such as polychlorazole mitigates this effect and improves appearance (Liu et al., 2018b).

Combining various hormones proves to be a more effective strategy for inducing parthenocarpic fruit formation, suggesting that plant parthenocarpy is governed by a complex network involving synergistic hormone interactions (Sharif et al., 2022). Auxin and gibberellin, produced in seeds post-fertilization, are crucial for kick-starting fruit development (He and Yamamuro, 2022). Moreover, applying these hormones externally can trigger the growth of seedless fruits, underscoring their importance in initiating this process. The interplay between auxin and gibberellin in regulating fruit set has been thoroughly researched; current understanding posits that auxin influences gibberellin activity (Serrani et al., 2007; Ozga et al., 2009). In Arabidopsis, either the auxin response induced by pollination or the auxin treatment can cause an increase in endogenous GA biosynthesis. However, adding gibberellins doesn’t immediately affect internal levels of auxins (Dorcey et al., 2009). In Arabidopsis *della* mutants, additional auxin does not intensify the seedless fruit trait—this suggests that effective auxin signaling requires activation of the GA-DELLA pathway (Fuentes et al., 2012). Research indicates that *SlARF7* serves as both a repressor of auxin signaling and a participant in the GA signaling pathway during tomato fruit set, playing a crucial dual role in regulating this process (de Jong et al., 2011). Molecular evidence shows that SlARF7 interacts with SlDELLA proteins to form a complex that negatively regulates its target genes (**Figure 1**) (Hu et al., 2018). Despite their collaborative roles in promoting parthenocarpy, auxins primarily encourage fruit expansion through increasing cell layers while gibberellins mainly enhance cell elongation—their coordinated action ensures proper development of the fruit (Serrani et al., 2008).

Cytokinins primarily facilitate parthenocarpy by stimulating cell division and increasing cell numbers (Matsuo et al., 2012). After flowering, the level of active cytokinins in unfertilized ovaries drops sharply, suggesting a strong link between early fruit development and ovarian cytokinin levels (Shinozaki et al., 2015). Some studies suggest that CPPU application boosts IAA levels in the ovary, thereby encouraging parthenocarpy. When ‘Pandex’ (parthenocarpic) and ‘Khira’ (non-parthenocarpic) varieties, along with their F1 hybrids, were treated with CPPU during bloom, a significant rise in IAA was noted in the ovaries of ‘Pandex’ (Kim et al., 1992). Additionally, CPPU treatment has been shown to increase endogenous GA_3_ levels (Chai et al., 2019). Experiments on tomatoes reveal that applying cytokinins to unfertilized ovaries promotes fruit growth; however, this effect is completely blocked when PAC—a gibberellin synthesis inhibitor—is used concurrently. This indicates that cytokinins may be crucial for regulating both cell division and gibberellin production in tomatoes (Matsuo et al., 2012; Ding et al., 2013). In pears, research shows that the expression pattern of transcription factor *PbRR9* involved in cytokinin signaling mirrors that of auxin synthesis gene *PbYUC4* but contrasts with abscisic acid synthesis gene *PbNECD6*’s pattern (Cong et al., 2020). Molecular evidence confirms PbARR9 directly interacts with both PbYUC4 and PbNECD6—suggesting cytokinins drive parthenocarpy by upregulating auxin-related genes while downregulating those associated with abscisic acid production (Cong et al., 2020).

The ethylene-insensitive *sletr1–1* mutant exhibits notably higher gibberellin levels in its fruit compared to wild-type tomatoes. Gibberellin synthesis gene *GA20ox3* transcripts rise, whereas those of the metabolism genes *GA2ox4* and *GA2ox5* fall (Shinozaki et al., 2015). Auxin levels, however, remain relatively stable. Treating *sletr1–1* plants with PAC, a gibberellin synthesis inhibitor, can prevent the development of seedless (parthenocarpic) fruit, suggesting that ethylene influences fruit set through the gibberellin pathway (**Table 1**) (Shinozaki et al., 2015). Following pollination, there is a marked decrease in the expression of genes associated with ethylene biosynthesis and signaling (Nitsch et al., 2009). Concurrently, abscisic acid (ABA) biosynthesis gene expression decreases while ABA degradation gene expression increases (Nitsch et al., 2009). Moreover, in tomato ABA mutants (*not/flc*), cells within the fruit are smaller; abscisic acid content drops without significant changes in auxin levels; yet ethylene release escalates (Nitsch et al., 2009). This suggests that ABA and ethylene may synergize with each other to regulate fruit set.

## Molecular mechanisms of MADS-box gene regulation in parthenocarpy

Angiosperms typically have four layers of floral organs in their buds, which are, from the outermost to the innermost, the sepals, petals, stamens, and pistils (López-Martínez et al., 2024). Each layer serves a distinct role in reproduction: sepals protect the bud; colorful petals attract pollinators; stamens produce pollen grains; and pistils, housing ovules, lead to seed production post-fertilization. The “ABC model” effectively illustrates how genes shape this conservative floral structure (Irish, 2017). Crucially, MADS-box genes are key regulators of floral organ and fruit development, with an expanding list identified in the regulation of parthenocarpy in tomatoes.

**Figure 2:**
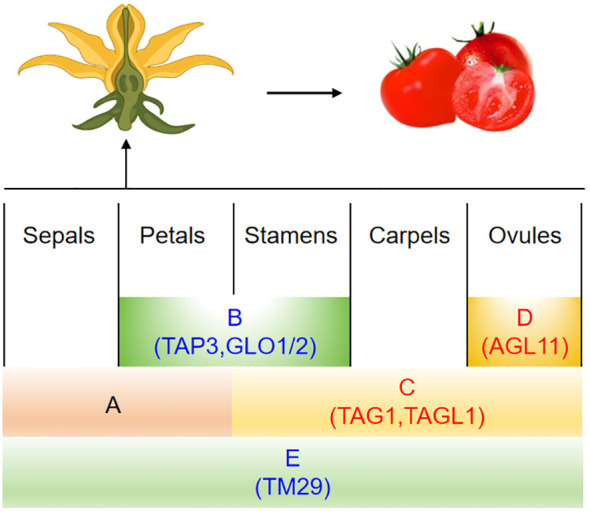
The ABCDE model genes of the MADS-box family are linked to tomato parthenocarpy. Genes marked in red indicate that overexpression results in parthenocarpy, while those in blue suggest that silencing the gene induces parthenocarpy.

The B-class genes within the MADS-box family play a crucial role in the development of petals and stamens in flowering plants (Kramer et al., 1998; Theißen et al., 2016). One such gene, *TAP3* (*TOMATO APETALA3*), demonstrates the significance of B-class genes. Mutations in *TAP3*, specifically the EMS mutant *sltap3* and plants with reduced *SlTAP3*, lead to notable transformations: anthers become sepals, stamens turn into carpels, pollen is aborted, and fruits without seeds are formed due to the expansion and enlargement of ovary wall cells (de Martino et al., 2006). Muntant of *carpelloid stamen and parthenocarpy (csp)* was identified parthenocarpy. It was a novel allelic mutation of *TAP3* (Li et al., 2024). Additionally, two related B-class genes, *SlGLO1* and *SlGLO2*, show higher expression levels during the early development stages of petals and stamens (Geuten and Irish, 2010). In plants where both *SlGLO1* and *SlGLO2* are suppressed, a similar transformation occurs as in *sltap3* mutants, with petals changing into sepals, stamens into carpels, and resulting in the production of seedless fruits (**Table 4**, **Figure 2**) (Geuten and Irish, 2010).

**Table 4:** Involvement of MADS-box gene family in tomato parthenocarpy.

Class of Homeotic Genes	Gene	ID	Cause	References
B Class	TAP3	Solyc04g081000	silencing	de Martino et al., 2006
B Class	GLO1	Solyc08g067230	silencing	Geuten and Irish, 2010
B Class	GLO2	Solyc06g059970	silencing	Geuten and Irish, 2010
C Class	TAG1	Solyc02g071730	overexpressing	Pnueli et al., 1994
C Class	TAGL1	Solyc07g055920	overexpressing	Vrebalov et al., 2009; Ribelles et al., 2019
D Class	AGL11	Solyc11g028020	overexpressing	Huang et al., 2017
E Class	TM29	Solyc02g089200	silencing	Ampomah-Dwamena et al., 2002
type II MIKCC	AGL6	Solyc01g093960	silencing	Klap et al., 2017
type II MIKCC	TM8	Solyc03g019710	silencing	Daminato et al., 2014

In *Arabidopsis*, the *AGAMOUS* gene (*AG*; a C-class gene) controls the development of stamens and carpels (Becker, 2003). In tomatoes, *TOMATO AGAMOUS* 1 (*TAG1*) is mainly expressed in these same floral parts (Pnueli et al., 1994). *TAG1* down-regulation transgenic plants were obtained by using the antisense technology. These plants showed a transformation of the third flower whorl into petal-like structures and produced parthenocarpic fruit (Pnueli et al., 1994). Additionally, another C-class gene known as *TOMATO AGAMOUS-LIKE 1* (*TAGL1*) influences tomato flowers, early fruit growth, and ripening stages. When *TAGL1* was overexpressed in plants driven by the 35S promoter, aberrant expression occurred in leaves and sepals. This resulted in premature closure of sepals during fruit development that hindered normal opening. The pollen was sterile and the ovary enlargement led to seedless fruits forming (Vrebalov et al., 2009). Furthermore, the *Alq-TAGL1* mutant also forms seedless fruits and matures early, confirming the involvement of *TAGL1* in fruit development (**Table 3**, **Figure 2**) (Ribelles et al., 2019).

The D-class gene *SlAGL11* (*TOMATO AGAMOUS-LIKE 11*) is expressed in both flowers and fruits, with particularly high levels during the early stages of fruit development. RNAi plants yield fruits with seeds; however, these seeds are smaller and the total fruit weight is reduced by 20%. No other significant phenotypic differences exist (Ocarez and Mejía, 2016; Huang et al., 2017). Overexpression of *SlAGL11* results in similar abnormalities as seen in *SlAGL1* overexpression, including distorted floral organ formation at initial bud stages, discoloration of sepals, a fleshy texture, and failure to open completely even when mature (Vrebalov et al., 2009). The carpels are entirely enclosed within the flower structure, leading to nonviable pollen and consequently seedless or minimally-seeded mature fruits that exhibit parthenocarpic traits (**Table 4**, **Figure 2**) (Huang et al., 2017).

The E-class gene *TM29* (*Tomato MADS 29*) plays a critical role in the development of tomato floral organs and fruit, particularly in sustaining meristematic tissue (Ampomah-Dwamena et al., 2002). In typical wild-type tomatoes, *TM29* is active within all four whorls of the flower structure. Using co-suppression and antisense strategies, scientists have reduced *TM29* expression, yielding genetically modified plants with distinct changes in their flowers: petals and stamens turn green instead of yellow, stamens and carpels become sterile—though these carpels may still produce seedless fruit (Ampomah-Dwamena et al., 2002). Another E-class member is *AGL6* (Dreni and Zhang, 2016). The *SlAGL6* (*AGAMOUS-like 6*) variant in tomatoes influences the creation of seedless fruits as well (Klap et al., 2017). An EMS-induced mutant of *slagl6* produced parthenocarpic fruits under high temperature conditions. The size and shape of the fruit and pollen fertility were not affected, making it a valuable tomato parthenocarpic germplasm (Klap et al., 2017). In-depth studies on this *slagl6* mutation revealed that during ovule maturation, the innermost endothelium layer fails to differentiate into integuments. However, by upregulating the cytochrome P450 cell proliferation regulator *SlKLUH*, there was excessive proliferation of the integument cells, which stimulated the expansion of the ovary wall, leading to non-fertilization-dependent seedless fruit formation (**Table 3**) (Gupta et al., 2021).

Additionally, the type II MIKC^C^ subfamily member *TM8* (*Tomato MADS 8*) is essential in tomato development. Overexpression of *TM8* results in abnormal stamen formation, reduced pollen viability, and altered expression of key flower genes including B, C, and E-class genes. Conversely, plants containing the repressed *TM8: SRDX* gene develop oval ovaries and seedless fruits (Daminato et al., 2014). The repercussions of *TM8’s* ectopic expression on reproductive structures underline its significance in the morphogenesis of tomato flowers and fruit. Therefore, by comprehensively regulating the MADS-box genes to control the phenotype of parthenocarpic fruit formation in tomatoes, changes also occur in the reproductive organs, such as floral organ homology (*TAG1, TAP3, GLO1, GLO2, TM29, TM8*), sepal thickening (*ALQ-TAGL1, TAG1, AGL11*), petal-like stamens (*TAG1*), and stamens with carpel-like features (*TAP3, GLO1, GLO2*) (**Table 4**, **Figure 2**).

## The other regulators involved in the regulation of parthenocarpy

Parthenocarpic fruit development is also influenced by *miRNAs*. The HD-Zip III transcription factor gene family is the target of *miRNA165/166*. Overexpressing *miRNA165* reduces HD-Zip III gene expression, causing abnormal carpels (Zhou et al., 2007). Suppressing *miRNA165/166* enhanced HD-Zip III transcription factors gene expression, leading to shorter stamens in transgenic plants. This indicates that HD-Zip III family genes were involved in anther development; their increased expression may also induce male sterility (Jia et al., 2015). SlHB15A is a member of the HD-Zip III family in tomato. Mutations in *SlHB15A* result in seedless tomatoes pointing to its inhibitory role on unfertilized fruit development. Moreover, wild-type plants’ fertility significantly drops under cold stress, unlike mutants of *SlHB15A*—which implies its potential for breeding parthenocarpic varieties (**Table 3**) (Clepet et al., 2021). The R2R3-MYB family comprises GAMYB-like factors; studies show that tomato’s *SlGAMYB1/2* are reduced by *miR159* during ovary development of parthenocarpic specimens (**Table 3**) (da Silva et al., 2017).

The transcriptional co-repressor TOPLESS has been shown to interact with Aux/IAA and ARF, key elements of the auxin signaling pathway (Szemenyei et al., 2008; Hao et al., 2014; He et al., 2021). In *Arabidopsis thaliana*, the *TPL* gene is active during pollen and ovule development. Examination of the *tpl-1* mutant showed that 43.8% of its ovules were degenerated while a further 41.8% turned out to be sterile, indicating that *At-TPL* plays a critical role in ovule formation as an essential gene in this process (Wei et al., 2015). In tomatoes, silencing *SlTPL1* via RNA interference (RNAi) demonstrated that reduced *SlTPL1* expression under emasculation and high temperatures results in the production of seedless fruits—a likely consequence of increased cytokinin levels in the ovaries (**Table 3**) (He et al., 2021).

The transgenic tomato obtained by introducing the ovary-specific promoter *TPRP* fused with the *root locus B (RolB)* gene into tomato was able to bear parthenocarpy under extreme high and low temperatures, with better yields and qualities than that of WT, and effectively addressed the issue of hollow fruit (Shabtai et al., 2007).

SlDOF10 is a gene coding a DNA-binding with one finger (DOF) transcription factor which is activated in unpollinated ovaries of the parthenocarpic plants. Down-regulation of *SlDOF10* activity led to the phenotype of parthenocarpic fruit set (Rojas-Gracia et al., 2019).

Mutant of *High Fruit Set under stress (HFS)* is parthenocarpic, meaning pollination is not required for fruit set. Tomato ‘*hfs*’ mutants do not affect normal growth phases but significantly boost both fruit set and yields under heat or saline conditions. Additionally, they offer advantages like improved flavor profiles and a higher sugar-acid balance favored during thermal stress. (**Table 3**) (Meco et al., 2019).

The *Alq* tomato mutant, which causes pre-anthesis ovary swelling and increased fruit setting, results in facultative parthenocarpic fruits and does not affect yield under salt stress conditions (Ribelles et al., 2019). At the same time, cell division in the *Alq* mutant ovary and the expression of genes related to the auxin and gibberellin signaling pathways are altered (Ribelles et al., 2019).

Research has found that the *SlSPL/HYDRA* gene is a key factor in initiating tomato gametogenesis. Mutations in the *HYDRA* gene in plants lead to incomplete development of male and female gametophytes. The development of the female gametophyte sac results in the formation of parthenocarpy fruits. Additionally, there are changes in the expression of genes related to gibberellin metabolism pathways (Rojas-Gracia et al., 2017).

Mutation of the F-box gene *hws* in tomato leads to the formation of facultative parthenocarpic fruit and reduces fertility, as well as changing leaf morphology. These phenotypes may be associated with the downregulation of auxin signaling pathway genes and the upregulation of *miRNA* expression in *hws* (**Table 3**) (Damayanti et al., 2019).

Finally, a novel *small parthenocarpic fruit and flower (spff)* mutant in the tomato was identified. The mutant showed both vegetative and reproductive phenotypes including altered axillary shoot development, male sterility, delayed flowering, and parthenocarpic production of small fruits (Takei et al., 2019).

## Conclusions and perspectives

In recent years, with the development of protected horticulture, the demand for seedless crop varieties in agriculture has been increasing. Although tomatoes have many parthenocarpy resources, their practical agricultural use is limited due to undesirable side effects. Consequently, there’s a pressing need to expedite the breeding of parthenocarpic tomato cultivars. Additionally, several issues require further clarification.

Omic research techniques are prevalent in plant science, with numerous studies utilizing RNA-seq, DAP-seq, and ChIP-seq to investigate parthenocarpic fruit development. However, there’s a scarcity of research on proteomics and metabolomics in seedless fruits. While exogenous hormone treatments can induce parthenocarpy, they often cause undesirable effects. Exploring new inducers using metabolomics and other omics technologies represents an exciting field for future research.

Research into the regulatory mechanisms of parthenocarpic fruit formation has primarily focused on internal factors; however, studies examining the interplay between external and internal influences remain scarce. Understanding how these mechanisms operate under adverse conditions continues to be a significant knowledge gap.

To date, tomato research on genes associated with parthenocarpy has concentrated on those regulating endogenous hormones or flower organ development through MADS regulators. Despite numerous gene discoveries related to this trait, molecular markers closely linked to it have yet to be identified. Developing such markers will aid in detecting, evaluating, and breeding for parthenocarpy in tomatoes and other fruit horticulture crops.

Finally, while much research has been conducted on the molecular mechanisms underlying tomato parthenocarpy, key genes involved remain unidentified. Investigating their specific genetic functions is crucial. Leveraging genetic traits and molecular markers to breed new varieties represents a vital direction for future work in this field.

## Author contributions

HG: Conceptualization, Data curation, Resources, Software, Validation, Visualization, Writing – original draft, Writing – review & editing, Formal Analysis, Investigation, Methodology. XY: Writing – review & editing, Data curation, Resources, Software, Validation, Writing – original draft. YL: Data curation, Methodology, Resources, Software, Visualization, Writing – review & editing. BX: Data curation, Resources, Software, Visualization, Writing – review & editing. XZ: Data curation, Resources, Software, Visualization, Writing – review & editing. CM: Data curation, Resources, Software, Visualization, Writing – review & editing. RX: Writing – review & editing. RC: Data curation, Funding acquisition, Project administration, Resources, Software, Supervision, Validation, Visualization, Writing – original draft, Writing – review & editing. YH: Conceptualization, Data curation, Funding acquisition, Project administration, Resources, Software, Supervision, Validation, Visualization, Writing – original draft, Writing – review & editing.

## References

[B1] Abad M. Monteiro A. A. (1989). The use of auxins for the production of greenhouse tomatoes in mild-winter conditions: a review. Sci. Hortic. 38, 167–192. doi: 10.1016/0304-4238(89)90064-2

[B2] Adamowski M. Friml J. (2015). Pin-dependent auxin transport: action, regulation, and evolution. Plant Cell 27, 20–32. doi: 10.1105/tpc.114.134874 25604445 PMC4330589

[B3] Ampomah-Dwamena C. Morris B. A. Sutherland P. Veit B. Yao J. (2002). Down-regulation of *TM29*, a tomato *SEPALLATA* homolog, causes parthenocarpic fruit development and floral reversion. Plant Physiol. 130, 605–617. doi: 10.1104/pp.005223 12376628 PMC166590

[B4] An J. Almasaud R. A. Bouzayen M. Zouine M. Chervin C. (2020). Auxin and ethylene regulation of fruit set. Plant Sci. 292, 110381. doi: 10.1016/j.plantsci.2019.110381 32005386

[B5] Becker A. (2003). The major clades of MADS-box genes and their role in the development and evolution of flowering plants. Mol. Phylogenet. Evol. 29, 464–489. doi: 10.1016/S1055-7903(03)00207-0 14615187

[B6] Beraldi D. Picarella M. E. Soressi G. P. Mazzucato A. (2004). Fine mapping of the parthenocarpic fruit (*pat*) mutation in tomato. Theor. Appl. Genet. 108, 209–216. doi: 10.1007/s00122-003-1442-6 14564391

[B7] Carrera E. Ruiz-Rivero O. Peres L. E. P. Atares A. Garcia-Martinez J. L. (2012). Characterization of the *procera* tomato mutant shows novel functions of the SLDELLA protein in the control of flower morphology, cell division and expansion, and the auxin-signaling pathway during fruit-set and development. Plant Physiol. 160, 1581–1596. doi: 10.1104/pp.112.204552 22942390 PMC3490602

[B8] Chai P. Dong S. Chai L. Chen S. Flaishman M. Ma H. (2019). Cytokinin-induced parthenocarpy of San Pedro type fig (*Ficus carica* L.) Main crop: explained by phytohormone assay and transcriptomic network comparison. Plant Mol. Biol. 99, 329–346. doi: 10.1007/s11103-019-00820-2 30656555

[B9] Chen D. Xu Y. Li J. Shiba H. Ezura H. Wang N. (2024). Erecta modulates seed germination and fruit development via auxin signaling in tomato. Int. J. Mol. Sci. 25, 4754. doi: 10.3390/ijms25094754 38731974 PMC11084166

[B10] Chen X. Tao J. Cao P. (2001). Types of parthenocarpy in horticultural crops. Biol. Bull. 35, 6–7. doi: 10.3969/j.issn.0006-3193.2001.09.003

[B11] Chen X. Zhang M. Tan J. Huang S. Wang C. Zhang H.. (2017). Comparative transcriptome analysis provides insights into molecular mechanisms for parthenocarpic fruit development in eggplant (*Solanum melongena L.*). PloS One 12, e179491. doi: 10.1371/journal.pone.0179491 PMC5467848 28604820

[B12] Clepet C. Devani R. S. Boumlik R. Hao Y. Morin H. Marcel F.. (2021). The miR166-SlHB15A regulatory module controls ovule development and parthenocarpic fruit set under adverse temperatures in tomato. Mol. Plant 14, 1185–1198. doi: 10.1016/j.molp.2021.05.005 33964458

[B13] Cong L. Wu T. Liu H. Wang H. Zhang H. Zhao G.. (2020). CPPU may induce gibberellin-independent parthenocarpy associated with *PBRR9* in ‘Dangshansu’ pear. Hortic. Res. 7, 68. doi: 10.1038/s41438-020-0285-5 32377358 PMC7192895

[B14] Cong L. Yue R. Wang H. Liu J. Zhai R. Yang J.. (2019). 2,4-D-induced parthenocarpy in pear is mediated by enhancement of GA_4_ biosynthesis. Physiol. Plant 166, 812–820. doi: 10.1111/ppl.12835 30203555

[B15] Costantini E. Landi L. Silvestroni O. Pandolfini T. Spena A. Mezzetti B. (2007). Auxin synthesis-encoding transgene enhances grape fecundity. Plant Physiol. 143, 1689–1694. doi: 10.1104/pp.106.095232 17337528 PMC1851826

[B16] Damayanti F. Lombardo F. Masuda J. Shinozaki Y. Ichino T. Hoshikawa K.. (2019). Functional disruption of the tomato putative ortholog of *HAWAIIAN* skirt results in facultative parthenocarpy, reduced fertility and leaf morphological defects. Front. Plant Sci. 10, 1234. doi: 10.3389/fpls.2019.01234 31681360 PMC6801985

[B17] Daminato M. Masiero S. Resentini F. Lovisetto A. Casadoro G. (2014). Characterization of *TM8*, a MADS-box gene expressed in tomato flowers. BMC Plant Biol. 14, 319. doi: 10.1186/s12870-014-0319-y 25433802 PMC4258831

[B18] da Silva E. M. Silva G. F. F. E. Bidoia D. B. Da Silva Azevedo M. de Jesus F. A. Ellen Pino L.. (2017). microRNA159-targeted SlGAMYB transcription factors are required for fruit set in tomato. Plant J. 92, 95–109. doi: 10.1111/tpj.13637 28715118

[B19] de Jong M. Wolters Arts M. Feron R. Mariani C. Vriezen W. H. (2009). The *Solanum lycopersicum* auxin response factor 7 (SlARF7) regulates auxin signaling during tomato fruit set and development. Plant J. 57, 160–170. doi: 10.1111/j.1365-313X.2008.03671.x 18778404

[B20] de Jong M. Wolters-Arts M. García-Martínez J. L. Mariani C. Vriezen W. H. (2011). The *Solanum lycopersicum* auxin response factor 7 (SlARF7) mediates cross-talk between auxin and gibberellin signalling during tomato fruit set and development. J. Exp. Bot. 62, 617–626. doi: 10.1093/jxb/erq293 20937732 PMC3003806

[B21] de Martino G. Pan I. Emmanuel E. Levy A. Irish V. F. (2006). Functional analyses of two tomato *APETALA3* genes demonstrate diversification in their roles in regulating floral development. Plant Cell. 18, 1833–1845. doi: 10.1105/tpc.106.042978 16844904 PMC1533988

[B22] Ding J. Chen B. Xia X. Mao W. Shi K. Zhou Y.. (2013). Cytokinin-induced parthenocarpic fruit development in tomato is partly dependent on enhanced gibberellin and auxin biosynthesis. PloS One 8, e70080. doi: 10.1371/journal.pone.0070080 23922914 PMC3726760

[B23] Dorcey E. Urbez C. Blázquez M. A. Carbonell J. Perez Amador M. A. (2009). Fertilization-dependent auxin response in ovules triggers fruit development through the modulation of gibberellin metabolism in Arabidopsis. Plant J. 58, 318–332. doi: 10.1111/j.1365-313X.2008.03781.x 19207215

[B24] Dreni L. Zhang D. (2016). Flower development: the evolutionary history and functions of the *AGL6* subfamily MADS-box genes. J. Exp. Bot. 67, 1625–1638. doi: 10.1093/jxb/erw046 26956504

[B25] El-Sharkawy I. Sherif S. El Kayal W. Jones B. Li Z. Sullivan A. J.. (2016). Overexpression of plum auxin receptor psltir1 in tomato alters plant growth, fruit development and fruit shelf-life characteristics. BMC Plant Biol. 16, 56. doi: 10.1186/s12870-016-0746-z 26927309 PMC4772300

[B26] Ezura K. Nomura Y. Ariizumi T. (2023). Molecular, hormonal, and metabolic mechanisms of fruit set, the ovary-to-fruit transition, in horticultural crops. J. Exp. Bot. 74, 6254–6268. doi: 10.1093/jxb/erad214 37279328

[B27] Ficcadenti N. Sestili1 S. Pandolfini T. Cirillo C. Rotino G. Spena A. (1999). Genetic engineering of parthenocarpic fruit development in tomato. Mol. Breed. 5, 463–470.

[B28] Fos M. Nuez F. García-Martínez J. L. (2000). The gene *pat-2*, which induces natural parthenocarpy, alters the gibberellin content in unpollinated tomato ovaries. Plant Physiol. 122, 471–480. doi: 10.1104/pp.122.2.471 10677440 PMC58884

[B29] Fos M. Proano K. Alabadı D. Nuez F. Carbonell J. Garcıa-Martınez J. L. (2003). Polyamine metabolism is altered in unpollinated parthenocarpic *pat-2* tomato ovaries. Plant Physiol. 131, 359–366. doi: 10.1104/pp.013037 12529543 PMC166815

[B30] Fuentes S. Ljung K. Sorefan K. Alvey E. Harberd N. P. Østergaard L. (2012). Fruit growth in *Arabidopsis* occurs via DELLA-dependent and DELLA-independent gibberellin responses. Plant Cell. 24, 3982–3996. doi: 10.1105/tpc.112.103192 23064323 PMC3517231

[B31] Gan Z. Feng Y. Wu T. Wang Y. Xu X. Zhang X.. (2019). Downregulation of the auxin transporter gene *SlPIN8* results in pollen abortion in tomato. Plant Mol. Biol. 99, 561–573. doi: 10.1007/s11103-019-00836-8 30734902

[B32] Gao S. Chu C. (2020). Gibberellin metabolism and signaling: targets for improving agronomic performance of crops. Plant Cell Physiol. 61, 1902–1911. doi: 10.1093/pcp/pcaa104 32761079 PMC7758032

[B33] García-Hurtado N. Carrera E. Ruiz-Rivero O. López-Gresa M. P. Hedden P. Gong F.. (2012). The characterization of transgenic tomato overexpressing gibberellin 20-oxidase reveals induction of parthenocarpic fruit growth, higher yield, and alteration of the gibberellin biosynthetic pathway. J. Exp. Bot. 63, 5803–5813. doi: 10.1093/jxb/ers229 22945942

[B34] Gaudin V. Jouanin L. (1995). Expression of *Agrobacterium rhizogenes* auxin biosynthesis genes in transgenic tobacco plants. Plant Mol. Biol. 28, 123–136. doi: 10.1007/BF00042044 7787177

[B35] Geuten K. Irish V. (2010). Hidden variability of floral homeotic B genes in *Solanaceae* provides a molecular basis for the evolution of novel functions. Plant Cell. 22, 2562–2578. doi: 10.1105/tpc.110.076026 20807882 PMC2947177

[B36] Gillaspy G. Ben-David H. Gruissem W. (1993). Fruits: a developmental perspective. Plant Cell. 5, 1439–1451. doi: 10.2307/3869794 12271039 PMC160374

[B37] Goetz M. Vivian-Smith A. Johnson S. D. Koltunow A. M. (2006). Auxin response factor 8 is a negative regulator of fruit initiation in Arabidopsis. Plant Cell. 18, 1873–1886. doi: 10.1105/tpc.105.037192 16829592 PMC1533983

[B38] Gorguet B. Eggink P. M. Ocaña J. Tiwari A. Schipper D. Finkers R.. (2008). Mapping and characterization of novel parthenocarpy QTLs in tomato. Theor. Appl. Genet. 6, 755–767. doi: 10.1007/s00122-007-0708-9 PMC2271080 18231773

[B39] Gorguet B. van Heusden A. W. Lindhout P. (2005). Parthenocarpic fruit development in tomato. Plant Biol. 7, 131–139. doi: 10.1055/s-2005-837494 15822008

[B40] Gou C. Zhu P. Meng Y. Yang F. Xu Y. Xia P.. (2022). Evaluation and genetic analysis of parthenocarpic germplasms in cucumber. Genes 13, 225. doi: 10.3390/genes13020225 35205270 PMC8872377

[B41] Gupta S. K. Barg R. Arazi T. (2021). Tomato *agamous-like 6* parthenocarpy is facilitated by ovule integument reprogramming involving the growth regulator *KLUH* . Plant Physiol. 185, 969–984. doi: 10.1093/plphys/kiaa078 33793903 PMC8133625

[B42] Hammes U. Z. Murphy A. S. Schwechheimer C. (2022). Auxin transporters-a biochemical view. C.S.H. Perspect. Biol. 14, a039875. doi: 10.1101/cshperspect.a039875 PMC8805647 34127449

[B43] Hao Y. Wang X. Li X. Bassa C. Mila I. Audran C.. (2014). Genome-wide identification, phylogenetic analysis, expression profiling, and protein-protein interaction properties of topless gene family members in tomato. J. Exp. Bot. 65, 1013–1023. doi: 10.1093/jxb/ert440 24399174 PMC3935560

[B44] Hazra P. Dutta A. K. Chatterjee P. (2010). Altered gibberellin and auxin levels in the ovaries in the manifestation of genetic parthenocarpy in tomato (*Solanum lycopersicum*). Curr. Sci. 99, 1439–1443.

[B45] He H. Yamamuro C. (2022). Interplays between auxin and GA signaling coordinate early fruit development. Hortic. Res. 19, uhab078. doi: 10.1093/hr/uhab078 PMC8955447 35043212

[B46] He M. Song S. Zhu X. Lin Y. Pan Z. Chen L.. (2021). *SlTPL1* silencing induces facultative parthenocarpy in tomato. Front. Plant Sci. 12, 672232. doi: 10.3389/fpls.2021.672232 34093628 PMC8174789

[B47] Hedden P. (2020). The current status of research on gibberellin biosynthesis. Plant Cell Physiol. 61, 1832–1849. doi: 10.1093/pcp/pcaa092 32652020 PMC7758035

[B48] Hu J. Israeli A. Ori N. Sun T. (2018). The interaction between DELLA and ARF/IAA mediates crosstalk between gibberellin and auxin signaling to control fruit initiation in tomato. Plant Cell. 30, 1710–1728. doi: 10.1105/tpc.18.00363 30008445 PMC6139683

[B49] Hu J. Li X. Sun T. (2023). Four class A AUXIN RESPONSE FACTORs promote tomato fruit growth despite suppressing fruit set. Nat. Plants. 9, 706–719. doi: 10.1038/s41477-023-01396-y 37037878 PMC10276352

[B50] Hua B. Wu J. Han X. Bian X. Xu Z. Sun C.. (2024). Auxin homeostasis is maintained by *sly-miR167-SlARF8A/B-SlGH3.4 f*eedback module in the development of locular and placental tissues of tomato fruits. New Phytol. 241, 1177–1192. doi: 10.1111/nph.19391 37985404

[B51] Huang B. Routaboul J. Liu M. Deng W. Maza E. Mila I.. (2017). Overexpression of the class D MADS-box gene *SlAGL11* impacts fleshy tissue differentiation and structure in tomato fruits. J. Exp. Bot. 68, 4869–4884. doi: 10.1093/jxb/erx303 28992179

[B52] Irish V. (2017). The ABC model of floral development. Curr. Biol. 27, R853–R909. doi: 10.1016/j.cub.2017.03.045 28898659

[B53] Israeli A. Schubert R. Man N. Teboul N. Serrani Yarce J. C. Rosowski E. E.. (2023). Modulating auxin response stabilizes tomato fruit set. Plant Physiol. 192, 2336–2355. doi: 10.1093/plphys/kiad205 37032117 PMC10315294

[B54] Ito T. Okada K. Fukazawa J. Takahashi Y. (2018). DELLA-dependent and -independent gibberellin signaling. Plant Signal. Behav. 13, e1445933. doi: 10.1080/15592324.2018.1445933 29485381 PMC5927702

[B55] Iwahori S. Tominaga S. Yamasaki T. (1988). Stimulation of fruit growth of kiwifruit, *Actinidia chinensis* planch., by N-(2-chloro-4-pyridyl)-N'-phenylurea, a diphenylurea-derivative cytokinin. Sci. Hortic. 35, 109–115. doi: 10.1016/0304-4238(88)90042-8

[B56] Jahn L. Hofmann U. Ludwig-Müller J. (2021). Indole-3-acetic acid is synthesized by the endophyte *Cyanodermella asteris* via a tryptophan-dependent and -independent way and mediates the interaction with a non-host plant. Int. J. Mol. Sci. 22, 2651. doi: 10.3390/ijms22052651 33800748 PMC7961953

[B57] Jia X. Ding N. Fan W. Yan J. Gu Y. Tang X. (2015). Functional plasticity of miR165/166 in plant development revealed by small tandem target mimic. Plant Sci. 233, 11–21. doi: 10.1016/j.plantsci.2014.12.020 25711809

[B58] Kai W. Fu Y. Wang J. Liang B. Li Q. Leng P. (2019). Functional analysis of *SlNCED1* in pistil development and fruit set in tomato (*Solanum lycopersicum* L.). Sci. Rep. 9, 16943. doi: 10.1038/s41598-019-52948-2 31729411 PMC6858371

[B59] Kim I. S. Okubo H. Fujieda K. (1992). Endogenous levels of IAA in relation to parthenocarpy in cucumber (*Cucumis sativus* L.). Sci. Hortic. 52, 1–2. doi: 10.1016/0304-4238(92)90002-T

[B60] Klap C. Yeshayahou E. Bolger A. M. Arazi T. Gupta S. K. Shabtai S.. (2017). Tomato facultative parthenocarpy results from *SlAGAMOUS-LIKE* 6 loss of function. Plant Biotechnol. J. 15, 634–647. doi: 10.1111/pbi.12662 27862876 PMC5399002

[B61] Knapp J. L. Bartlett L. J. Osborne J. L. (2017). Re-evaluating strategies for pollinator-dependent crops: how useful is parthenocarpy? J. Appl. Ecol. 54, 1171–1179. doi: 10.1111/1365-2664.12813 28781379 PMC5516152

[B62] Kramer E. M. Dorit R. L. Irish V. F. (1998). Molecular evolution of genes controlling petal and stamen development: duplication and divergence within the *APETALA3* and *PISTILLATA* MADS-box gene lineages. Genetics 149, 765–783. doi: 10.1093/genetics/149.2.765 9611190 PMC1460198

[B63] Krecek P. Skupa P. Libus J. Naramoto S. Tejos R. Frim J.. (2009). The PIN-FORMED (PIN) protein family of auxin transporters. Genome Biol. 10, 249. doi: 10.1186/gb-2009-10-12-249 20053306 PMC2812941

[B64] Lemaire-Chamley M. Petit J. Garcia V. Just D. Baldet P. Germain V.. (2005). Changes in transcriptional profiles are associated with early fruit tissue specialization in tomato. Plant Physiol. 139, 750–769. doi: 10.1104/pp.105.063719 16183847 PMC1255993

[B65] Leyser O. (2018). Auxin signaling. Plant Physiol. 176, 465–479. doi: 10.1104/pp.17.00765 28818861 PMC5761761

[B66] Li S. Wei K. Zhang L. Yu N. Lu F. Wang X.. (2024). Fine mapping and candidate gene validation of tomato gene *Carpelloid Stamen and Parthenocarpy* (*CSP*). Horticulturae 10, 403. doi: 10.3390/horticulturae10040403

[B67] Lin S. George W. L. Splittstoesser W. E. (1984). Expression and inheritance of parthenocarpy in 'Severianin' tomato. J. Hered. 75, 62–66. doi: 10.1093/oxfordjournals.jhered.a109867

[B68] Lin Z. Arciga-Reyes L. Zhong S. Alexander L. Hackett R. Wilson I.. (2008). SlTPR1, a tomato tetratricopeptide repeat protein, interacts with the ethylene receptors NR and LeETR1, modulating ethylene and auxin responses and development. J. Exp. Bot. 59, 4271–4287. doi: 10.1093/jxb/ern276 19036844 PMC2639023

[B69] Liu J. Zhai R. Liu F. Zhao Y. Wang H. Liu L.. (2018a). Melatonin induces parthenocarpy by regulating genes in gibberellin pathways of ‘Starkrimson’ pear (*Pyrus communis* L.). Front. Plant Scie. 9, 946. doi: 10.3389/fpls.2018.00946 PMC6040045 30022992

[B70] Liu L. Wang Z. Liu J. Liu F. Zhai R. Zhu C.. (2018b). Histological, hormonal and transcriptomic reveal the changes upon gibberellin-induced parthenocarpy in pear fruit. Hortic. Res. 5, 1. doi: 10.1038/s41438-017-0012-z 29423231 PMC5798812

[B71] Liu S. Zhang Y. Feng Q. Qin L. Pan C. Lamin-Samu A. T.. (2018c). Tomato auxin response factor 5 regulates fruit set and development via the mediation of auxin and gibberellin signaling. Sci. Rep. 8, 2971. doi: 10.1038/s41598-018-21315-y 29445121 PMC5813154

[B72] Livne S. Lor V. S. Nir I. Eliaz N. Aharoni A. Olszewski N. E.. (2015). Uncovering DELLA-independent gibberellin responses by characterizing new tomato *procera* mutants. Plant Cell. 27, 1579–1594. doi: 10.1105/tpc.114.132795 26036254 PMC4498196

[B73] López-Martínez A. M. Magallón S. von Balthazar M. Schönenberger J. Sauquet H. Chartier M. (2024). Angiosperm flowers reached their highest morphological diversity early in their evolutionary history. New Phytol. 241, 1348–1360. doi: 10.1111/nph.19389 38029781 PMC10952840

[B74] Luo J. Zhou J. Zhang J. (2018). Aux/IAA gene family in plants: molecular structure, regulation, and function. Int. J. Mol. Sci. 19, 259. doi: 10.3390/ijms19010259 29337875 PMC5796205

[B75] Luo W. Li Y. Sun Y. Lu L. Zhao Z. Zhou J.. (2021). Comparative RNA-seq analysis reveals candidate genes associated with fruit set in pumpkin. Sci. Hortic. 288, 110255. doi: 10.1016/j.scienta.2021.110255

[B76] Ma Q. Dong C. (2021). Regulatory functions and molecular mechanisms of ethylene receptors and receptor-associated proteins in higher plants. Plant Growth Regul. 93, 39–52. doi: 10.1007/s10725-020-00674-5

[B77] Mäkilä R. Wybouw B. Smetana O. Vainio L. Solé-Gil A. Lyu M.. (2023). Gibberellins promote polar auxin transport to regulate stem cell fate decisions in cambium. Na. Plants 9, 631–644. doi: 10.1038/s41477-023-01360-w PMC10119023 36997686

[B78] Mao Z. Yu Q. Zhen W. Guo J. Hu Y. Gao Y.. (2002). Expression of *ipt* gene driven by tomato fruit specific promoter and its effects on fruit development of tomato. Chin. Sci. Bull. 47, 928–933. doi: 10.1360/02tb9208

[B79] Mariotti L. Picciarelli P. Lombardi L. Ceccarelli N. (2011). Fruit-set and early fruit growth in tomato are associated with increases in indoleacetic acid, cytokinin, and bioactive gibberellin contents. J. Plant Growth Regul. 30, 405–415. doi: 10.1007/s00344-011-9204-1

[B80] Maroto J. V. Miguel A. Lopez-Galarza S. San Bautista A. Pascual B. Alagarda J.. (2005). Parthenocarpic fruit set in triploid watermelon induced by CPPU and 2,4-D applications. Plant Growth Regul. 45, 209–213. doi: 10.1007/s10725-005-3992-x

[B81] Martí C. Orzáez D. Ellul P. Moreno V. Carbonell J. Granell A. (2007). Silencing of *DELLA* induces facultative parthenocarpy in tomato fruits. Plant J. 52, 865–876. doi: 10.1111/j.1365-313X.2007.03282.x 17883372

[B82] Martínez-Bello L. Moritz T. López-Díaz I. (2015). Silencing C_19_-GA 2-oxidases induces parthenocarpic development and inhibits lateral branching in tomato plants. J. Exp. Bot. 66, 5897–5910. doi: 10.1093/jxb/erv300 26093022 PMC4566981

[B83] Mata C. I. Fabre B. Parsons H. T. Hertog M. L. A. T. Van Raemdonck G. Baggerman G.. (2018). Ethylene receptors, CTRs and EIN2 target protein identification and quantification through parallel reaction monitoring during tomato fruit ripening. Front. Plant Scie. 9, 1626. doi: 10.3389/fpls.2018.01626 PMC6235968 30467512

[B84] Matsuo S. Kikuchi K. Fukuda M. Honda I. Imanishi S. (2012). Roles and regulation of cytokinins in tomato fruit development. J. Exp. Bot. 63, 5569–5579. doi: 10.1093/jxb/ers207 22865911 PMC3444270

[B85] Matsuo S. Miyatake K. Endo M. Urashimo S. Kawanishi T. Negoro S.. (2020). Loss of function of the *Pad-1* aminotransferase gene, which is involved in auxin homeostasis, induces parthenocarpy in *Solanaceae* plants. Proc. Natl. Acad. Sci. U.S.A. 117, 12784–12790. doi: 10.1073/pnas.2001211117 32461365 PMC7293652

[B86] Mazzucato A. Testa G. Biancari T. Soressi G. (1999). Effect of gibberellic acid treatments, environmental conditions, and genetic background on the expression of the *parthenocarpic fruit* mutation in tomato. Protoplasma 208, 18–25. doi: 10.1007/BF01279071

[B87] McGinnis K. M. Thomas S. G. Soule J. D. Strader L. C. Zale J. M. Sun T. P.. (2003). The Arabidopsis *SLEEPY1* gene encodes a putative F-box subunit of an SCF E3 ubiquitin ligase. Plant Cell. 15, 1120–1130. doi: 10.1105/tpc.010827 12724538 PMC153720

[B88] Meco V. Egea I. Albaladejo I. Campos J. F. Morales B. Ortíz-Atienza A.. (2019). Identification and characterisation of the tomato parthenocarpic mutant *high fruit set under stress* (*hfs*) exhibiting high productivity under heat and salt stress. Ann. Appl. Biol. 174, 166–178. doi: 10.1111/aab.12486

[B89] Mesihovic A. Iannacone R. Firon N. Fragkostefanakis S. (2016). Heat stress regimes for the investigation of pollen thermotolerance in crop plants. Plant Reprod. 29, 93–105. doi: 10.1007/s00497-016-0281-y 27016360

[B90] Mezzetti B. Landi L. Pandolfini T. Spena A. (2004). The *defH9-iaaM* auxin-synthesizing gene increases plant fecundity and fruit production in strawberry and raspberry. BMC Biotechnol. 4, 4. doi: 10.1186/1472-6750-4-4 15113427 PMC394336

[B91] Mignolli F. Vidoz M. L. Mariotti L. Lombardi L. Picciarelli P. (2015). Induction of gibberellin 20-oxidases and repression of gibberellin 2β-oxidases in unfertilized ovaries of *entire* tomato mutant, leads to accumulation of active gibberellins and parthenocarpic fruit formation. Plant Growth Regul. 75, 415–425. doi: 10.1007/s10725-014-0002-1

[B92] Miller C. O. Skoog F. Okumura F. S. von Saltza M. H. Strong F. M. (1955). Structure and synthesis of kinetin. J. Am. Chem. Soc 11, 2662–2663. doi: 10.1021/ja01614a108

[B93] Mockaitis K. Estelle M. (2008). Auxin receptors and plant development: a new signaling paradigm. Annu. Rev. Cell Dev. Biol. 24, 55–80. doi: 10.1146/annurev.cellbio.23.090506.123214 18631113

[B94] Mok D. W. Mok M. C. (2001). Cytokinin metabolism and action. Annu. Rev. Plant Biol. 52, 89–118. doi: 10.1146/annurev.arplant.52.1.89 11337393

[B95] Molesini B. Dusi V. Pennisi F. Pandolfini T. (2020). How hormones and MADS-box transcription factors are involved in controlling fruit set and parthenocarpy in tomato. Genes 11, 1441. doi: 10.3390/genes11121441 33265980 PMC7760363

[B96] Molesini B. Pandolfini T. Rotino G. L. Dani V. Spena A. (2009). *Aucsia* gene silencing causes parthenocarpic fruit development in tomato. Plant Physiol. 149, 534–548. doi: 10.1104/pp.108.131367 18987210 PMC2613741

[B97] Mounet F. Moing A. Kowalczyk M. Rohrmann J. Petit J. Garcia V.. (2012). Down-regulation of a single auxin efflux transport protein intomato induces precocious fruit development. J. Exp. Bot. 63, 4901–4917. doi: 10.1093/jxb/ers167 22844095 PMC3427993

[B98] Mubarok S. Jadid N. Widiastuti A. Derajat Matra D. Budiarto R. Lestari F. W.. (2023). Parthenocarpic tomato mutants, *iaa9-3* and *iaa9-5*, show plant adaptability and fruiting ability under heat-stress conditions. Front. Plant Sci. 14, 1090774. doi: 10.3389/fpls.2023.1090774 36938002 PMC10014533

[B99] Murase K. Hirano Y. Sun T. Hakoshima T. (2008). Gibberellin-induced DELLA recognition by the gibberellin receptor GID1. Nature 456, 459–463. doi: 10.1038/nature07519 19037309

[B100] Nguyen H. N. Lai N. Kisiala A. B. Emery R. J. N. (2021). Isopentenyltransferases as master regulators of crop performance: their function, manipulation, and genetic potential for stress adaptation and yield improvement. Plant Biotechnol. J. 19, 1297–1313. doi: 10.1111/pbi.13603 33934489 PMC8313133

[B101] Nitsch L. M. Oplaat C. Feron R. Ma Q. Wolters-Arts M. Hedden P.. (2009). Abscisic acid levels in tomato ovaries are regulated by *LeNCED1* and *SlCYP707A1* . Planta 229, 1335–1346. doi: 10.1007/s00425-009-0913-7 19322584

[B102] Niu S. He Y. Yan S. Sun Z. Cai R. Zhang Y. (2024). Histological, transcriptomic, and gene functional analyses reveal the regulatory events underlying gibberellin-induced parthenocarpy in tomato. Hortic. Plant J. 10, 156–170. doi: 10.1016/j.hpj.2023.01.002

[B103] Ocarez N. Mejía N. (2016). Suppression of the D-class MADS-box *AGL11* gene triggers seedlessness in fleshy fruits. Plant Cell Rep. 35, 239–254. doi: 10.1007/s00299-015-1882-x 26563346

[B104] Okabe Y. Yamaoka T. Ariizumi T. Ushijima K. Kojima M. Takebayashi Y.. (2019). Aberrant stamen development is associated with parthenocarpic fruit set through up-regulation of gibberellin biosynthesis in tomato. Plant Cell Physiol. 60, 38–51. doi: 10.1093/pcp/pcy184 30192961

[B105] Olimpieri I. Siligato F. Caccia R. Soressi G. P. Mazzucato A. (2007). Tomato fruit set driven by pollination or by the parthenocarpic fruit allele are mediated by transcriptionally regulated gibberellin biosynthesis. Planta 226, 877–888. doi: 10.1007/s00425-007-0533-z 17503074

[B106] Ozga J. A. Reinecke D. M. Ayele B. T. Ngo P. Nadeau C. Wickramarathna A. D. (2009). Developmental and hormonal regulation of gibberellin biosynthesis and catabolism in pea fruit. Plant Physiol. 150, 448–462. doi: 10.1104/pp.108.132027 19297588 PMC2675736

[B107] Pan C. Yang D. Zhao X. Liu Y. Li M. Ye L.. (2021). PIF4 negatively modulates cold tolerance in tomato anthers via temperature-dependent regulation of tapetal cell death. Plant Cell. 33, 2320–2339. doi: 10.1093/plcell/koab120 34009394 PMC8364245

[B108] Pandolfini T. Rotino G. L. Camerini S. Defez R. Spena A. (2002). Optimisation of transgene action at the post-transcriptional level: high quality parthenocarpic fruits in industrial tomatoes. BMC Biotechnol. 2, 1. doi: 10.1186/1472-6750-2-1 11818033 PMC65046

[B109] Pascual L. Blanca J. M. Canizares J. Nuez F. (2009). Transcriptomic analysis of tomato carpel development reveals alterations in ethylene and gibberellin synthesis during *pat3/pat4* parthenocarpic fruit set. BMC Plant Biol. 9, 67. doi: 10.1186/1471-2229-9-67 19480705 PMC2700107

[B110] Picken A. (1984). A review of pollination and fruit set in the tomato (*Lycopersicon esculentum* Mill.). J. Hortic. 59, 1–13. doi: 10.1080/00221589.1984.11515163

[B111] Pnueli L. Hareven D. Rounsley S. D. Yanofsky M. F. Lifschitz E. (1994). Isolation of the tomato *AGAMOUS* gene *TAG1* and analysis of its homeotic role in transgenic plants. Plant Cell. 6, 163–173. doi: 10.1080/00221589.1984.11515163 7908549 PMC160424

[B112] Qiu S. (1984). Parthenocarpy and hormone. Plant Physiol. Commun. 1, 5. doi: 10.13592/j.cnki.ppj.1984.02.001

[B113] Ren Z. Li Z. Miao Q. Yang Y. Deng W. Hao Y. (2011). The auxin receptor homologue in *Solanum lycopersicum* stimulates tomato fruit set and leaf morphogenesis. J. Exp. Bot. 62, 2815–2826. doi: 10.1093/jxb/erq455 21266497

[B114] Ribelles C. García-Sogo B. Yuste-Lisbona F. J. Atarés A. Castañeda L. Capel C.. (2019). *Alq* mutation increases fruit set rate and allows the maintenance of fruit yield under moderate saline conditions. J. Exp. Bot. 70, 5731–5744. doi: 10.1093/jxb/erz342 31328220

[B115] Rodrigo M. J. García-Martínez J. L. (1998). Hormonal control of parthenocarpic ovary growth by the apical shoot in pea. Plant Physiol. 116, 511–518. doi: 10.1104/pp.116.2.511 9490755 PMC35108

[B116] Rojas-Gracia P. Roque E. Medina M. López-Martín M. J. Cañas L. A. Beltrán J. P.. (2019). The DOF transcription factor SlDOF10 regulates vascular tissue formation during ovary development in tomato. Front. Plant Sci. 10, 216. doi: 10.3389/fpls.2019.00216 30863420 PMC6399211

[B117] Rojas-Gracia P. Roque E. Medina M. RoChina M. Hamza R. Angarita-Díaz M. P.. (2017). The parthenocarpic *hydra* mutant reveals a new function for a *SPOROCYTELESS-like* gene in the control of fruit set in tomato. New Phytol. 214, 1198–1212. doi: 10.1111/nph.14433 28134991

[B118] Rotino G. L. Perri E. Zottini M. Sommer H. Spena A. (1997). Genetic engineering of parthenocarpic plants. Nat. Biotechnol. 15, 1398–1401. doi: 10.1038/nbt1297-1398 9415894

[B119] Schijlen E. G. W. M. de Vos C. H. R. Martens S. Jonker H. H. Rosin F. M. Molthoff J. W.. (2007). RNA interference silencing of chalcone synthase, the first step in the flavonoid biosynthesis pathway, leads to parthenocarpic tomato fruits. Plant Physiol. 144, 1520–1530. doi: 10.1104/pp.107.100305 17478633 PMC1914118

[B120] Schwartz S. H. Tan B. C. Gage D. A. Zeevaart J. A. D. McCarty D. R. (1997). Specific oxidative cleavage of carotenoids by VP14 of maize. Science 276, 1872–1874. doi: 10.1126/science.276.5320.1872 9188535

[B121] Serrani J. C. Carrera E. Ruiz-Rivero O. Gallego-Giraldo L. Peres L. E. P. García-Martínez J. L. (2010). Inhibition of auxin transport from the ovary or from the apical shoot induces parthenocarpic fruit-set in tomato mediated by gibberellins. Plant Physiol. 153, 851–862. doi: 10.1104/pp.110.155424 20388661 PMC2879769

[B122] Serrani J. C. Fos M. Atarés A. García-Martínez J. L. (2007). Effect of gibberellin and auxin on parthenocarpic fruit growth induction in the cv Micro-tom of tomato. J. Plant Growth Regul. 26, 211–221. doi: 10.1007/s00344-007-9014-7

[B123] Serrani J. C. Ruiz Rivero O. Fos M. García-Martínez J. L. (2008). Auxin-induced fruit-set in tomato is mediated in part by gibberellins. Plant J. 56, 922–934. doi: 10.1111/j.1365-313X.2008.03654.x 18702668

[B124] Shabtai S. Salts Y. Kaluzky G. Barg R. (2007). Improved yielding and reduced puffiness under extreme temperatures induced by fruit-specific expression of *rolB* in processing tomatoes. Theor. Appl. Genet. 114, 1203–1209. doi: 10.1007/s00122-007-0511-7 17279365

[B125] Sharif R. Su L. Chen X. Qi X. (2022). Hormonal interactions underlying parthenocarpic fruit formation in horticultural crops. Hortic. Res. 9, uhab024. doi: 10.1093/hr/uhab024 35031797 PMC8788353

[B126] Shinozaki Y. Hao S. Kojima M. Sakakibara H. Ozeki Iida Y. Zheng Y.. (2015). Ethylene suppresses tomato (*Solanum lycopersicum*) fruit set through modification of gibberellin metabolism. Plant J. 83, 237–251. doi: 10.1111/tpj.12882 25996898

[B127] Su L. Rahat S. Ren N. Kojima M. Takebayashi Y. Sakakibara H.. (2021). Cytokinin and auxin modulate cucumber parthenocarpy fruit development. Sci. Hortic. 282, 110026. doi: 10.1016/j.scienta.2021.110026

[B128] Szemenyei H. Hannon M. Long J. A. (2008). TOPLESS mediates auxin-dependent transcriptional repression during Arabidopsis embryogenesis. Science 319, 1384–1386. doi: 10.1126/science.1151461 18258861

[B129] Takei H. Shinozaki Y. Yano R. Kashojiya S. Hernould M. Chevalier C.. (2019). Corrigendum: loss-of-function of a tomato receptor-like kinase impairs male fertility and induces parthenocarpic fruit set. Front. Plant Sci. 10, 403. doi: 10.3389/fpls.2019.00403 31040856 PMC6477066

[B130] Takisawa R. Nakazaki T. Nunome T. Fukuoka H. Kataoka K. Saito H.. (2018). The parthenocarpic gene *Pat-k* is generated by a natural mutation of *SlAGL6* affecting fruit development in tomato (*Solanum lycopersicum* L.). BMC Plant Biol. 18, 72. doi: 10.1186/s12870-018-1285-6 29699487 PMC5921562

[B131] Teale W. D. Paponov I. A. Palme K. (2006). Auxin in action: signalling, transport and the control of plant growth and development. Nat. Rev. Mol. Cell Biol. 7, 847–859. doi: 10.1038/nrm2020 16990790

[B132] Theißen G. Melzer R. Rümpler F. (2016). MADS-domain transcription factors and the floral quartet model of flower development: linking plant development and evolution. Dev 143, 3259–3271. doi: 10.1242/dev.134080 27624831

[B133] Ueguchi-Tanaka M. Ashikari M. Nakajima M. Itoh H. Katoh E. Kobayashi M.. (2005). Gibberellin insensitive *dwarf1* encodes a soluble receptor for gibberellin. Nature 437, 693–698. doi: 10.1038/nature04028 16193045

[B134] Ueta R. Abe C. Watanabe T. Sugano S. S. Ishihara R. Ezura H.. (2017). Rapid breeding of parthenocarpic tomato plants using CRISPR/Cas9. Sci. Rep. 7, 507. doi: 10.1038/s41598-017-00501-4 28360425 PMC5428692

[B135] Ulmasov T. Liu Z. B. Hagen G. Guilfoyle T. J. (1995). Composite structure of auxin response elements. Plant Cell. 7, 1611–1623. doi: 10.1105/tpc.7.10.1611 7580254 PMC161020

[B136] Varoquaux F. Blanvillain R. Delseny M. Gallois P. (2000). Less is better: new approaches for seedless fruit production. Trends Biotechnol. 18, 233–242. doi: 10.1016/S0167-7799(00)01448-7 10802558

[B137] Vivian-Smith A. Koltunow A. M. (1999). Genetic analysis of growth-regulator-induced parthenocarpy in Arabidopsis. Plant Physiol. 121, 437–452. doi: 10.1104/pp.121.2.437 10517835 PMC59406

[B138] Vrebalov J. Pan I. L. Arroyo A. J. M. McQuinn R. Chung M. Poole M.. (2009). Fleshy fruit expansion and ripening are regulated by the tomato *SHATTERPROOF* gene *TAGL1* . Plant Cell. 21, 3041–3062. doi: 10.1105/tpc.109.066936 19880793 PMC2782289

[B139] Vriezen W. H. Feron R. Maretto F. Keijman J. Mariani C. (2008). Changes in tomato ovary transcriptome demonstrate complex hormonal regulation of fruit set. New Phytol. 177, 60–76. doi: 10.1111/j.1469-8137.2007.02254.x 18028300

[B140] Wang H. Jones B. Li Z. Frasse P. Delalande C. Regad F.. (2005). The tomato AUX/IAA transcription factor IAA9 is involved in fruit development and leaf morphogenesis. Plant Cell. 17, 2676–2692. doi: 10.1105/tpc.105.033415 16126837 PMC1242265

[B141] Wang H. Wu T. Liu J. Cong L. Zhu Y. Zhai R.. (2020). PbGA20ox_2_ regulates fruit set and induces parthenocarpy by enhancing GA_4_ content. Front. Plant Sci. 11, 113. doi: 10.3389/fpls.2020.00113 32133025 PMC7039935

[B142] Wei B. Zhang J. Pang C. Yu H. Guo D. Jiang H.. (2015). The molecular mechanism of SPOROCYTELESS/NOZZLE in controlling Arabidopsis ovule development. Cell Res. 25, 121–134. doi: 10.1038/cr.2014.145 25378179 PMC4650584

[B143] Yang S. F. Hoffman N. E. (1984). Ethylene biosynthesis and its regulation in higher plants. Ann. Rev. Plant Physiol. 35, 155–189. doi: 10.1146/annurev.pp.35.060184.001103

[B144] Yin Z. Malinowski R. Ziółkowska A. Sommer H. Plcader W. Malepszy S. (2006). The DefH9-iaaM-containing construct efficiently induces parthenocarpy in cucumber. Cell. Mol. Biol. Lett. 11, 279–290. doi: 10.2478/s11658-006-0024-4 16847572 PMC6275954

[B145] Zazimalova E. Murphy A. S. Yang H. Hoyerova K. Hosek P. (2010). Auxin transporters-why so many? Cold Spring Harb. Perspec. Biol. 2, a1552. doi: 10.1101/cshperspect.a001552 PMC2829953 20300209

[B146] Zhang J. Chen R. Xiao J. Qian C. Wang T. Li H.. (2007). A single-base deletion mutation in *SlIAA9* gene causes tomato (*Solanum lycopersicum*) *entire* mutant. J. Plant Res. 120, 671–678. doi: 10.1007/s10265-007-0109-9 17955175

[B147] Zhang S. Gu X. Shao J. Hu Z. Zhu L. (2021). Auxin metabolism is involved in fruit set and early fruit development in the parthenocarpic tomato "R35-p". Front. Plant Sci. 12, 671713. doi: 10.3389/fpls.2021.671713 34408758 PMC8365229

[B148] Zhang T. (2013). Expression analysis of genes and preliminary mapping of major QTL related to parthenocarpy of cucumber (Nanjing Agricultural University).

[B149] Zhao Y. (2010). Auxin biosynthesis and its role in plant development. Annu. Rev. Plant Biol. 61, 49–64. doi: 10.1146/annurev-arplant-042809-112308 20192736 PMC3070418

[B150] Zhou G. Kubo M. Zhong R. Demura T. Ye Z. (2007). Overexpression of *miR165* affects apical meristem formation, organ polarity establishment and vascular development in Arabidopsis. Plant Cell Physiol. 48, 391–404. doi: 10.1093/pcp/pcm008 17237362

[B151] Zhou S. Yang Y. Zou M. Tao T. Tang X. Wang Y.. (2023). The molecular mechanism of eggplant parthenocarpy revealed through a combined analysis of the transcriptome and the metabolome. Ind. Crops Prod. 193, 116168. doi: 10.1016/j.indcrop.2022.116168

[B152] Zhu H. Lang D. Mo S. Liu Y. (2007). Studies on the cytological mechanism of plant seedless fruit. Hebei J. Forest. Orchard Res. 22, 259–261. doi: 1007-4961(2007)03-0259-03

[B153] Zhu H. Zhu B. Shao Y. Wang X. Lin X. Xie Y.. (2006). Tomato fruit development and ripening are altered by the silencing of *LeEIN2* gene. J. Integr. Plant Biol. 48, 1478–1485. doi: 10.1111/j.1744-7909.2006.00366.x

